# Identifying Intrinsically Disordered Protein Regions through a Deep Neural Network with Three Novel Sequence Features

**DOI:** 10.3390/life12030345

**Published:** 2022-02-26

**Authors:** Jiaxiang Zhao, Zengke Wang

**Affiliations:** College of Electronic Information and Optical Engineering, Nankai University, Tianjin 300350, China; wangzk@mail.nankai.edu.cn

**Keywords:** intrinsically disordered proteins, the persistent entropy, the probabilities associated with two and three consecutive amino acids, VGG19

## Abstract

The fast, reliable, and accurate identification of IDPRs is essential, as in recent years it has come to be recognized more and more that IDPRs have a wide impact on many important physiological processes, such as molecular recognition and molecular assembly, the regulation of transcription and translation, protein phosphorylation, cellular signal transduction, etc. For the sake of cost-effectiveness, it is imperative to develop computational approaches for identifying IDPRs. In this study, a deep neural structure where a variant VGG19 is situated between two MLP networks is developed for identifying IDPRs. Furthermore, for the first time, three novel sequence features—i.e., persistent entropy and the probabilities associated with two and three consecutive amino acids of the protein sequence—are introduced for identifying IDPRs. The simulation results show that our neural structure either performs considerably better than other known methods or, when relying on a much smaller training set, attains a similar performance. Our deep neural structure, which exploits the VGG19 structure, is effective for identifying IDPRs. Furthermore, three novel sequence features—i.e., the persistent entropy and the probabilities associated with two and three consecutive amino acids of the protein sequence—could be used as valuable sequence features in the further development of identifying IDPRs.

## 1. Introduction

Protein regions which lack stable three-dimensional structures are referred to as intrinsically disordered regions (IDPRs) [1]. In recent years, it has come to be recognized more and more that IDPRs have a huge impact on many important physiological processes [2,3], such as molecular recognition and molecular assembly, the regulation of transcription and translation, protein phosphorylation, cellular signal transduction, etc. [4,5,6]. Furthermore, some human diseases, such as certain types of cancer, Parkinson’s disease, and cardiovascular disease [7,8,9], have been found to be linked with IDPRs. However, the experimental methods used to identify IDPRs are usually expensive and time-consuming [10]. Thus, the fast, reliable, and accurate identification of IDPRs by computational methods is a valuable complement to experimental studies.

There are many computational methods for identifying IDPRs. These methods can be divided into three categories: (1) Physicochemical-based methods, such as FoldIndex [11], GlobPlot [12], IUPred [13], FoldUnfold [14], and IsUnstruct [15], which rely on the amino acid physiochemical properties for identifying disorder. (2) Machine learning-based methods—for instance, DISvgg [16], RFPR-IDP [17], IDP-Seq2Seq [18], SPOT-Disorder [19], SPOT-Disorder2 [20], DISOPRED3 [21], SPINE-D [22], ESpritz [23], BVDEA [10], POODLE-S [24], RONN [25], and PONDRs [26]—which treat the identification of IDRs as labeling each amino acid of a protein sequence or as a classification problem. (3) Meta methods, including MFDp [27], MetaPrDOS [28], and Meta-Disorder predictor [29], which fuse multiple predictors to yield the final prediction for IDPRs.

While all of the above methods have contributed to the development of the field, there are still some new features that have not been discovered. Because of the interaction between amino acids, the question of how to describe them is key to improving predictions based on protein sequences.

In this paper, we develop a deep neural structure composed of a variant VGG19 [30], where the variant VGG19 is situated between two multilayer perceptron (MLP) networks for identifying IDPRs. In the variant VGG19, we erase the fully connected (FC) layers of VGG19 but preserve the other parts of the VGG19 structure and related parameters. In comparison with ResNet, the parameters of VGGNet could be easily manipulated. The MLP network consists of an input layer, hidden layers, and an output layer. The MLPs are employed for transforming the features into the formats suitable for serving as the inputs of the variant VGG19 and classification network, respectively. Compared with our previous DISpre algorithm [31] and DISvgg algorithm [16], we introduce VGG19 as a part of the network instead of as a single MLP network, and additionally use one VGG19 instead of ten VGG16. Moreover, to further improve the performance of prediction, we introduce new features for prediction. For the first time, three sequence features, which are the persistent entropy based on the persistent homology and the probabilities associated with two and three consecutive amino acids of the protein sequence (PCAA2, PCAA3), are introduced for identifying IDPRs. These three novel sequence features together with those used in [32]—i.e., two sequence features, seven physicochemical propensities, and three propensities of amino acids, as well as twenty evolutionary features—are used as the inputs for our neural structure. The simulation results obtained for two blind testing sets, R80 [25] and MXD494 [33], show that our neural structure either performs considerably better than other well-known methods [17,20] or, when relying on a much smaller training set (DIS1616) compared to the one used in [18], attains a similar performance.

## 2. Datasets and Input Features

In this section, the datasets used in this paper for training and blind testing are presented. The features extracted from the training dataset are depicted. In particular, we introduce three novel features, which are used for the first time for identifying IDPRs. These three novel features are persistent entropy based on persistent homology, PCAA2, and PCAA3.

### 2.1. Datasets

The dataset DIS1616 from the DisProt [34] (accessed on June 2020) is employed for training and cross validating, while the datasets R80 [25] and MXD494 [33] are used for blind testing. The training dataset DIS1616 consists of 1616 protein sequences which contain 182,316 disordered and 706,362 ordered amino acids. The dataset DIS1616 is randomly split into two subsets: DIS1450 and DIS166. They contain 1450 protein sequences and 166 protein sequences and are used for training and testing, respectively. The blind testing dataset R80 has 78 protein sequences, in which there are 3566 disordered and 29,243 ordered amino acids. There are 494 protein sequences in the blind testing dataset, MXD494, among which 44,087 disordered and 152,414 ordered amino acids are presented.

### 2.2. Input Features Used for the Identification of IDPRs

The features fed to our neural structure for identifying IDPRs can be summarized as five sequence features, seven physiochemical propensities, and three propensities of amino acids, as well as twenty evolutionary features of the given protein sequence. Of these five sequence features, persistent entropy based on persistent homology, PCAA2, and PCAA3 are, for the first time, introduced for identifying IDPRs. The remaining two sequence features are the Shannon entropy and topological entropy [32]. Topological entropy is used to depict the complexity of the protein sequence. The seven physiochemical properties of the amino acids are steric parameter, polarizability, volume, hydrophobicity, isoelectric point, helix, and sheet probability, as illustrated in the reference [35]. Three propensities of the amino acids are Remark 465, Deleage/Roux, and Bfactor(2STD), which are derived from the GlobPlot NAR paper [12]. Twenty evolutionary features can be determined through the Position-Specific Substitution Matrix (PSSM) [36], which is computed using the Position-Specific Iterative Basic Local Alignment Search Tool (PSI-BLAST) [37].

#### 2.2.1. The Computation of Persistent Entropy

In this section, we will to briefly illustrate the procedure used for computing the persistent homology as well as its persistent entropy from the given protein sequence. More information related to the computation of the persistent homology and its persistent entropy can be found in [38,39].

Given a protein sequence $\hat{w}=w_{1}\cdots w_{L}$ of length *L*, we choose a sliding window of odd length *N* (N<L) to extract *N* consecutive amino acids from $\hat{w}$. For simplicity, we first transform $\hat{w}$ into a sequence of size L+N−1 through appending (N−1)/2 amino acids to both ends of $\hat{w}$. The (N−1)/2 appended amino acids at both ends are identical to either the first or last amino acid of the protein sequence $\hat{w}$ . Thus, utilizing a sliding window of size *N*, we can slice the transformed $\hat{w}$ of size L+N−1 into *L* amino acid subsequences $\beta_{j}=w_{j}\cdots w_{j+(N-1)}$ with 1≤j≤L. To compute the persistent entropy of $\beta_{j}$, we need to map each amino acid $w_{m}$ in $\beta_{j}$ to a set of points, which leads us to define

(1) $$I_{1}\left(m\right)=\sum_{\Phi_{1}\left(k\right)\in \Upsilon_{1}}k\delta (w_{m}-\Phi_{1}\left(k\right))\;\;\;\;\mathrm{for}\;\;\;1\leq m\leq N$$

where the value for *k* is 1≤k≤20 and δ(·) is the delta function. We use a one to one correspondence to represent the set of amino acid symbols as:

(2) $$\begin{array}{c} \Upsilon_{1}≜\{\Phi_{1}\left(1\right)\;,\;\ldots \;,\;\Phi_{1}\left(20\right)\}\\ \;=\{A,R,N,D,C,Q,E,G,H,I,L,K,M,F,P,S,T,W,Y,V\}\;. \end{array}$$

Thus, each amino acid symbol $w_{m}$ with 1≤m≤N in $\beta_{j}=w_{j}\cdots w_{j+(N-1)}$ (1≤j≤L) is mapped to $\left(x_{m}^{\beta_{j}}\;,y_{m}^{\beta_{j}}\;,z_{m}^{\beta_{j}}\right)\in R^{3}$, where we have:

(3) $$\begin{array}{cc} x_{m}^{\beta_{j}}= & \cos\frac{2\pi }{20}I_{1}\left(m\right)\\ y_{m}^{\beta_{j}}= & \sin\frac{2\pi }{20}I_{1}\left(m\right)\\ z_{m}^{\beta_{j}}= & \frac{1}{N-1}\left(m-1\right) \end{array}\;.$$

We use $x_{m}^{\beta_{j}}=\cos\frac{2\pi }{20}I_{1}\left(m\right)$ and $y_{m}^{\beta_{j}}=\sin\frac{2\pi }{20}I_{1}\left(m\right)$ to project different amino acids to different positions on the axis. $x_{m}^{\beta_{j}}$ and $y_{m}^{\beta_{j}}$ are combined with $z_{m}^{\beta_{j}}$; then, all amino acids in $\beta_{j}$ are projected to different positions on the axis. Thus, we can map each amino acid $w_{m}$ for 1≤m≤N in $\beta_{j}=w_{j}\cdots w_{j+(N-1)}$ (1≤j≤L) to a unique element in the set of ${\left\{\left(x_{m}^{\beta_{j}}\;,y_{m}^{\beta_{j}}\;,z_{m}^{\beta_{j}}\right)\right\}}_{m=1}^{N}$ in $R^{3}$ through Equations (1)–(3).

The persistent entropy of V associated with ${\left\{\left(x_{m}^{\beta_{j}}\;,y_{m}^{\beta_{j}}\;,z_{m}^{\beta_{j}}\right)\right\}}_{m=1}^{N}$ can be computed as

(4) $$\begin{array}{cc} E(V) & =-\sum_{i=1}^{n}p_{i}\log_{2}p_{i}\;,\;\;\;\;p_{i}=\frac{\varepsilon_{e_{i}}-\varepsilon_{s_{i}}}{S_{L}}\;,\;\;\;\;S_{L}=\sum_{i=1}^{n}l_{i}\;, \end{array}$$

where V denotes a filtration with its associated persistence diagram $dgm\left(V\right)=\{\left(\varepsilon_{s_{i}}\;,\varepsilon_{e_{i}}\right)\;:\;\;1\leq i\leq n\}$ (we assume $\varepsilon_{s_{i}}<\varepsilon_{e_{i}}$ for all 1≤i≤n). We have $L={\left\{l_{i}=\varepsilon_{e_{i}}-\varepsilon_{s_{i}}\right\}}_{i=1}^{n}$. A filtration V of the simplicial complex $VR(\beta_{j},K)$ (K>0) associated with ${\left\{\left(x_{m}^{\beta_{j}}\;,y_{m}^{\beta_{j}}\;,z_{m}^{\beta_{j}}\right)\right\}}_{m=1}^{N}$ is obtained through increasing the parameter values 0≤ε≤K—i.e.,

(5) $$\phi =VR\left(\beta_{j},0\right)\subseteq VR\left(\beta_{j},\varepsilon_{1}\right)\subseteq \cdots \subseteq VR\left(\beta_{j},\varepsilon_{l}\right)=VR\left(\beta_{j},K\right)$$

with $0<\varepsilon_{1}<\cdots <\varepsilon_{l}=K$. In Equation (5), the simplicial complex $VR(\beta_{j},K)$ (K>0) is chosen to be the Vietoris Rips complex of $\beta_{j}$, which is defined as:

(6) $$\begin{array}{c} VR(\beta_{j},\varepsilon )=\{\sigma \subset {\left\{\left(x_{m}^{\beta_{j}}\;,y_{m}^{\beta_{j}}\;,z_{m}^{\beta_{j}}\right)\right\}}_{m=1}^{N}\;:\\ \;B_{\varepsilon }\left(\left(x_{m}^{\beta_{j}}\;,y_{m}^{\beta_{j}}\;,z_{m}^{\beta_{j}}\right)\right)\cap B_{\varepsilon }\left(\left(x_{l}^{\beta_{j}}\;,y_{l}^{\beta_{j}}\;,z_{l}^{\beta_{j}}\right)\right)\neq \phi \\ \;\forall \;\;\left(x_{m}^{\beta_{j}}\;,y_{m}^{\beta_{j}}\;,z_{m}^{\beta_{j}}\right)\;,\;\;\left(x_{l}^{\beta_{j}}\;,y_{l}^{\beta_{j}}\;,z_{l}^{\beta_{j}}\right)\in \sigma \} \end{array}$$

where $B_{\varepsilon }\left(\left(x_{m}^{\beta_{j}}\;,y_{m}^{\beta_{j}}\;,z_{m}^{\beta_{j}}\right)\right)$ is the ball centered at $\left(x_{m}^{\beta_{j}}\;,y_{m}^{\beta_{j}}\;,z_{m}^{\beta_{j}}\right)$ with the radius ε≥0 . Given a filtration V defined by (5), a barcode in the *k*-dimensional persistence with endpoints $\left[\varepsilon_{s},\varepsilon_{e}\right)$ corresponds to a *k*-dimensional hole that appears at filtration time $\varepsilon_{s}$ and remains until filtration time $\varepsilon_{e}$. The set of bars $\left[\varepsilon_{s},\varepsilon_{e}\right)$, representing the birth and death times of homology classes, is called the persistence barcode B(V) for the filtration V of (5). Analogously, the set of points $\left(\varepsilon_{s},\varepsilon_{e}\right)\in R^{2}$ is called the persistence diagram dgm(V) of the filtration V of (5). The persistent entropy of each amino acid $w_{m}$ for 1≤m≤N in $\beta_{j}=w_{j}\cdots w_{j+(N-1)}$ (1≤j≤L) is therefore equal to the persistent entropy E(V) of V associated with ${\left\{\left(x_{m}^{\beta_{j}}\;,y_{m}^{\beta_{j}}\;,z_{m}^{\beta_{j}}\right)\right\}}_{m=1}^{N}$.

#### 2.2.2. The Computation of the Features Using the Probabilities Associated with the Protein Sequence

The probability associated with two and three consecutive amino acids of the protein sequence depends on the probability of each amino acid occurring in the observed protein, which depends on the protein sequence length and the number of each individual amino acids in the protein sequence. We put all amino acids from all proteins in DIS1616 together and, based on this set, calculate the probabilities associated with two and three consecutive amino acids of the protein sequence. Consider the given protein sequence $\hat{w}=w_{1}\cdots w_{L}$. For convenience, we define two sets:

(7) $$\begin{array}{c} \Upsilon_{2}≜\{\Phi_{2}\left(1\right)\;,\;\ldots \;,\;\Phi_{2}\left(400\right)\}\\ \;≜\{AA,AR,AN,AD,AC\;,\;\ldots \;,\;VS,VT,VW,VY,VV\} \end{array}$$

(8) $$\begin{array}{c} \Upsilon_{3}≜\{\Phi_{3}\left(1\right)\;,\;\ldots \;,\;\Phi_{3}\left(8000\right)\}\\ \;≜\{AAA,AAR,AAN,AAD,AAC\;,\;\ldots \;,\;VVS,VVT,VVW,VVY,VVV\} \end{array}$$

which represent all the possible combinations of two or three consecutive amino acids in this protein sequence. Two novel features introduced in this paper are:

(9) $$\begin{array}{cc} \; & H_{2}=\left[H_{2}\left(1\right)\;,\ldots \;,H_{2}\left(L\right)\right] \end{array}$$

(10) $$\begin{array}{cc} \; & H_{3}=\left[H_{3}\left(1\right)\;,\ldots \;,H_{3}\left(L\right)\right] \end{array}$$

which can be derived from the probability features $P_{2}$ and $P_{3}$, respectively, associated with two or three consecutive amino acids of the protein sequence $\hat{w}=w_{1}\cdots w_{L}$. Using the notation δ(·) function, $H_{2}\left(j\right)$ and $H_{3}\left(j\right)$ in Equations (9) and (10) for 1≤j≤L can, respectively, be computed using:

(11) $$\begin{array}{cc} \; & \;H_{2}\left(j\right)=\left\{\begin{array}{cccc} \; & N_{2}\left(I_{2}\left(j\right)\right)\;\; & \; & j=1\\ \; & \frac{1}{2}\left(N_{2}\left(I_{2}\left(j-1\right)\right)+N_{2}\left(I_{2}\left(j\right)\right)\right)\; & \; & 2\leq j\leq L-1,\\ \; & N_{2}\left(I_{2}\left(j-1\right)\right)\; & \; & j=L \end{array}\right. \end{array}$$

(12) $$\begin{array}{cc} \; & \;H_{3}\left(j\right)=\left\{\begin{array}{cccc} \; & N_{3}\left(I_{3}\left(j\right)\right)\; & \; & j=1\\ \; & \frac{1}{2}\left(N_{3}\left(I_{3}\left(j-1\right)\right)+N_{3}\left(I_{3}\left(j\right)\right)\right)\; & \; & j=2\\ \; & \frac{1}{3}\left(N_{3}\left(I_{3}\left(j-2\right)\right)+N_{3}\left(I_{3}\left(j-1\right)\right)+N_{3}\left(I_{3}\left(j\right)\right)\right)\; & \; & 3\leq j\leq L-2\\ \; & \frac{1}{2}\left(N_{3}\left(I_{3}\left(j-2\right)\right)+N_{3}\left(I_{3}\left(j-1\right)\right)\right)\; & \; & j=L-1\\ \; & N_{3}\left(I_{3}\left(j-2\right)\right)\; & \; & j=L\;. \end{array}\right. \end{array}$$

In view of (7) and (8), functions $I_{2}\left(j\right)$ and $I_{3}\left(j\right)$ in (11) and (12) are defined as:

(13) $$\begin{array}{cc} \; & \begin{array}{c} I_{2}\left(j\right)=\sum_{\Phi_{2}\left(k\right)\in \Upsilon_{2}}k\delta \left(w_{j}-\Phi_{2}\left(k\right)\right)\begin{array}{cc} \; & \end{array} \end{array} \end{array}$$

(14) $$\begin{array}{cc} \; & \begin{array}{c} I_{3}\left(j\right)=\sum_{\Phi_{3}\left(k\right)\in \Upsilon_{3}}k\delta \left({\bar{w}}_{j}-\Phi_{3}\left(k\right)\right)\begin{array}{cc} \; & \end{array} \end{array} \end{array}$$

where $w_{j}$ and ${\bar{w}}_{j}$, respectively, represent $w_{j}w_{j+1}\left(1\leq j\leq L-1\right)$ and $w_{j}w_{j+1}w_{j+2}\left(1\leq j\leq L-2\right)$. It is easy to verify $1\leq I_{2}\left(j\right)\leq 400$ and $1\leq I_{2}\left(l\right)\leq 8000$ for 1≤j≤L−1 and 1≤l≤L−2. Functions $N_{2}\left(\cdot \right)$ and $N_{3}\left(\cdot \right)$ in (11) and (12) defined over the sets {1,…,400} and {1,…,8000}, respectively, are scaled probability features $P_{2}$ and $P_{3}$, with

(15) $$\begin{array}{cc} \; & N_{2}\left(k\right)=\;\;\left\{\begin{array}{cc} \frac{P_{2}\left(k\right)-\min_{P_{2}\left(k\right)\in P_{2}}\left\{P_{2}\left(k\right)\right\}}{\Delta_{2}} & \begin{array}{cc} \; & \mathrm{if}\begin{array}{cc} & \begin{array}{cc} \; & \Delta_{2}\neq 0 \end{array} \end{array} \end{array}\\ 1 & \begin{array}{cc} \; & \mathrm{if}\begin{array}{cc} & \begin{array}{cc} \; & \Delta_{2}=0 \end{array} \end{array} \end{array} \end{array}\right.1\leq k\leq 400 \end{array}$$

(16) $$\begin{array}{cc} \; & N_{3}\left(k\right)=\;\;\left\{\begin{array}{cc} \frac{P_{3}\left(k\right)-\min_{P_{3}\left(k\right)\in P_{3}}\left\{P_{3}\left(k\right)\right\}}{\Delta_{3}} & \begin{array}{cc} \; & \mathrm{if}\begin{array}{cc} & \begin{array}{cc} \; & \Delta_{3}\neq 0 \end{array} \end{array} \end{array}\\ 1 & \begin{array}{cc} \; & \mathrm{if}\begin{array}{cc} & \begin{array}{cc} \; & \Delta_{3}=0 \end{array} \end{array} \end{array} \end{array}\right.1\leq k\leq 8000 \end{array}$$

where we have $\Delta_{2}=\max_{P_{2}\left(k\right)\in P_{2}}\left\{P_{2}\left(k\right)\right\}-\min_{P_{2}\left(k\right)\in P_{2}}\left\{P_{2}\left(k\right)\right\}$ and $\Delta_{3}=\max_{P_{3}\left(k\right)\in P_{3}}\left\{P_{3}\left(k\right)\right\}-\min_{P_{3}\left(k\right)\in P_{3}}\left\{P_{3}\left(k\right)\right\}$. The probability features $P_{2}$ and $P_{3}$ associated with two and three consecutive amino acids of the protein sequence, respectively, are equal to:

(17) $$\begin{array}{cc} \; & P_{2}≜\left\{P_{2}\left(1\right)\;,\ldots \;,P_{2}\left(400\right)\right\}≜\left\{\frac{S_{2}\left(1\right)}{\sum_{k=1}^{400}S_{2}\left(k\right)}\;,\ldots \;,\frac{S_{2}\left(400\right)}{\sum_{k=1}^{400}S_{2}\left(k\right)}\right\} \end{array}$$

(18) $$\begin{array}{cc} \; & P_{3}≜\left\{P_{3}\left(1\right)\;,\ldots \;,P_{3}\left(8000\right)\right\}≜\left\{\frac{S_{3}\left(1\right)}{\sum_{k=1}^{8000}S_{3}\left(k\right)}\;,\ldots \;,\frac{S_{3}\left(8000\right)}{\sum_{k=1}^{8000}S_{3}\left(k\right)}\right\}\; \end{array}$$

In (17) and (18), we have:

(19) $$\begin{array}{cc} S_{2}\left(k\right) & =\sum_{\hat{w}\in \Omega }M_{\Phi_{2}\left(k\right)}^{\hat{w}}\;\;\;\;\;\;\;\;\;1\leq k\leq 400 \end{array}$$

(20) $$\begin{array}{cc} S_{3}\left(k\right) & =\sum_{\hat{w}\in \Omega }M_{\Phi_{3}\left(k\right)}^{\hat{w}}\;\;\;\;\;\;\;\;\;1\leq k\leq 8000 \end{array}$$

where we assume that the set of the protein sequences is denoted by Ω (in this paper, we have Ω=DIS1616). For a given protein sequence $\hat{w}=w_{1}\cdots w_{L}$, the functions $M_{\Phi_{2}\left(k\right)}^{\hat{w}}$ and $M_{\Phi_{3}\left(k\right)}^{\hat{w}}$, which, respectively, count the total number of occurrences of a particular combination of two or three consecutive amino acids in $\hat{w}$, are equal to:

(21) $$\begin{array}{cc} M_{\Phi_{2}\left(k\right)}^{\hat{w}} & =\sum_{j=1}^{L-1}\delta \left(w_{j}-\Phi_{2}\left(k\right)\right)\;\;\;\;\;\;\;\;1\leq k\leq 400 \end{array}$$

(22) $$\begin{array}{cc} M_{\Phi_{3}\left(k\right)}^{\hat{w}} & =\sum_{j=1}^{L-2}\delta \left({\bar{w}}_{j}-\Phi_{3}\left(k\right)\right)\;\;\;\;\;\;\;\;1\leq k\leq 8000 \end{array}$$

where $w_{j}$ and ${\bar{w}}_{j}$, respectively, represent $w_{j}w_{j+1}\left(1\leq j\leq L-1\right)$ for 1≤j≤L−1 and $w_{j}w_{j+1}w_{j+2}\left(1\leq j\leq L-2\right)$ for 1≤j≤L−2.

#### 2.2.3. Pre-Processing the Data Extracted from the Protein Sequences

In this section, we illustrate how to compute the input of our deep neural network, which is composed of 35 features derived from a protein sequence. Of these 35 features, there are twenty evolutionary features which are determined through the PSSM [36] computed through the PSI-BLAST [37]. Seven physiochemical properties of the amino acids are steric parameter, polarizability, volume, hydrophobicity, isoelectric point, helix, and sheet probability, which can be obtained from the paper [35]. Three propensities of the amino acids are Remark 465, Deleage/Roux, and Bfactor (2STD), as detailed in the GlobPlot NAR paper [12]. The other two features used to measure the complexity of the protein sequence are Shannon entropy and topological entropy [32].

Given a protein sequence $\hat{w}=w_{1}\cdots w_{L}$ of length *L*, we choose a sliding window of odd size *N* (N<L) to extract *N* consecutive amino acids. Then, for these amino acids in the sliding window, we compute the evolutionary features, physiochemical properties, and propensities, as defined in the previous paragraph. These thirty computed feature values of amino acids in the sliding window are averaged and the averaged results are used to represent the feature values of the amino acid in the center of the sliding window. For simplicity, we first transform $\hat{w}$ into a sequence of size L+N−1 through appending (N−1)/2 zeros to the both ends of the protein sequence. With this sliding window of size *N*, we also compute the Shannon and topological entropy through the procedure from Equations (1)–(14), as described in the paper [32], as well as the persistent entropy defined in (4). Thus, for each $w_{j}$ with 1≤j≤L in the protein sequence $\hat{w}=w_{1}\cdots w_{L}$, we can combine it with a 33×L feature matrix

(23) $$v_{j}=\left[\begin{array}{cccc} m_{1,j} & m_{2,j} & ... & m_{33,j} \end{array}\right]$$

where $m_{k,j}$ for 1≤k≤20 , 21≤k≤27 , 28≤k≤30 and 31≤k≤33, respectively, align to a 20-dimensional PSSM of the evolutionary information [36,37], seven physiochemical properties [35], three propensities of amino acids from the paper [12], and three entropies (Shannon, topological [32], and persistent entropy). We also use Equations (11) and (12) to compute two novel features $H_{2}\left(j\right)$ and $H_{3}\left(j\right)$ (1≤j≤L) that are associated with two or three consecutive amino acids of the protein sequence and set $m_{34,j}=H_{2}\left(j\right)$ and $m_{35,j}=H_{3}\left(j\right)$. Finally, we modify the 33×L feature matrix defined in (23) to a 35×L feature matrix:

(24) $$F=[\begin{array}{cccc} x_{1} & x_{2} & ... & x_{L} \end{array}]$$

with

(25) $$x_{j}={\left[\begin{array}{ccc} v_{j} & m_{34,j} & m_{35,j} \end{array}\right]}^{T}\;\;\;\;\;\;\;\;1\leq j\leq L$$

where $v_{j}$ (1≤j≤L) is defined in (23). The input to our deep neural network is $x_{j}$ (1≤j≤L), as defined in (25).

## 3. The Structure of Our Neural Network and Training Procedure

In this section, we develop a deep neural structure composed of a variant VGG19, where the variant VGG19 is situated between two MLP networks used for identifying IDPRs. Then, we introduce the process of training the deep neural network.

### 3.1. The Structure of Our Deep Neural Network

**Figure 1:**
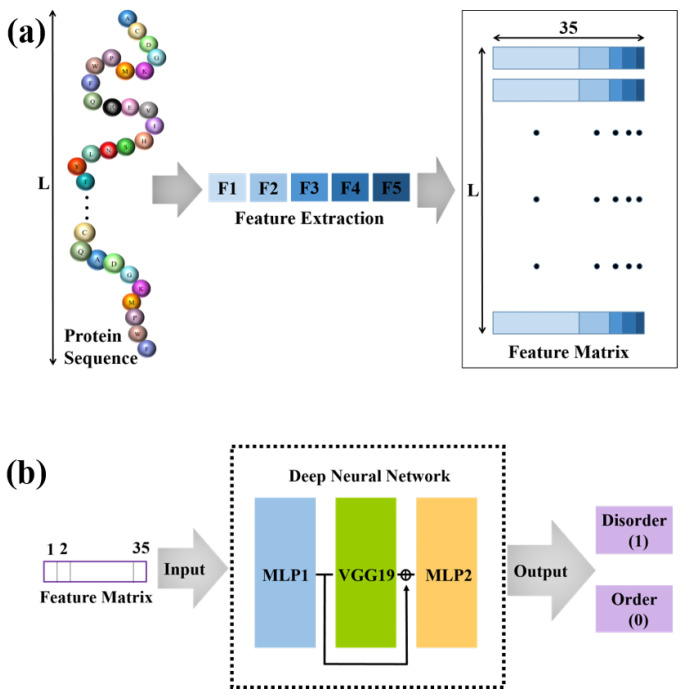
The overall framework for the prediction of intrinsically disordered proteins. (**a**) We extract five types of features from the protein sequence and obtain the feature matrix with 35 features for each amino acid. (**b**) The obtained feature matrix is input into the deep neural network. The output can be used to predict IDPRs.

The overall architecture of our model, as shown in Figure 1, is based on a variant VGG19 in cascade with two MLP networks, with the variant VGG19 being situated between two MLP networks. In the variant VGG19, we erase the fully connected (FC) layers of VGG19 but preserve the remaining VGG19 structure and its associated weights and biases.

**Figure 2:**
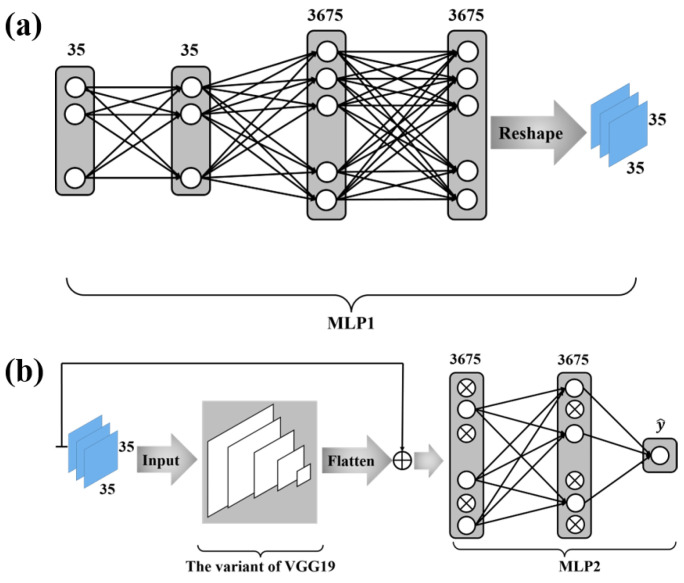
The deep neural network configuration. (**a**) is the first part of the deep neural network configuration. The function of MLP1 is to convert the protein sequence features into a mode suitable for VGG19 input. (**b**) is the second part of the deep neural network configuration. We use a variant of VGG19 for further feature extraction and MLP2 for classification. In MLP2, a dropout algorithm is used.

Figure 2a depicts the structure of the MLP network whose outputs are fed as the inputs to the variant VGG19 . This MLP network with two hidden layers takes each column (i.e., 35×1 features) defined in (25) as its input and yields a 1×3675 vector as its output. The 1×3675 output vector of this MLP network is then mapped to a 35×35×3 matrix through the reshape function of Keras, and this 35×35×3 matrix is fed as the input to the variant VGG19. The two hidden layers contain 35 and 3675 neurons, respectively. The activation functions of neurons in this MLP are the rectified linear unit (ReLU).

The output of the variant VGG19 is a 1×3675 vector. As shown in Figure 2b, the skip connection is employed, where the sum of the output from the variant VGG19 and the output from the MLP network connecting to the features defined in (25) is fed as the input to a novel MLP network. This MLP network contains one hidden layer with 3675 neurons, whose activation functions are chosen to be the ReLU. The output layer has only 1 neuron with the sigmoid function as its activation function—i.e.,

(26) $$\sigma \left(z_{i}\right)=\frac{1}{1+e^{-z_{i}}}$$

where $z_{i}$ (1≤i≤L) is the output of this sigmoid function and the index *i* is the *i*-th amino acid in the protein sequence $\hat{w}=w_{1}\cdots w_{L}$. The dropout algorithm [40] with a dropout percentage of 50% is employed for this MLP network.

The total loss function of our model for a package of size *m* (i.e., the number of amino acids used in each iteration during the training) is therefore defined as:

(27) $$L=\frac{1}{m}\sum_{i=1}^{m}-\left[y_{i}\ln\left(a_{i}\right)+\left(1-y_{i}\right)\ln\left(1-a_{i}\right)\right]\;.$$

In Equation (27), the predicted probability $a_{i}$ of the output $y_{i}=1$ is equal to:

(28) $$a_{i}≜\sigma \left(z_{i}\right)=\frac{1}{1+e^{-z_{i}}}\;$$

where $y_{i}$ is equal to either 1, suggesting that the *i*-th amino acid is disordered, or to 0, implying that it is ordered.

### 3.2. Training Procedure

In this section, we present the process of training the deep neural network developed in the previous section. The training dataset we use in this paper is DIS1450 from the DisProt [34]. We put all amino acids from all proteins in DIS1450 together and, based on this set, randomly divide them into packages of 128 amino acids. The training procedure is as follows: For each amino acid in a given package, we use the deep neural network constructed above to calculate the predicted probability defined in the Equation (28). When we have calculated all predicted probabilities for this given package, we can use the Equation (27) to estimate the average loss for the package. This computed averaged loss of the package is used to update the weights and biases of our network via a stochastic gradient descent (SGD) algorithm [41], where the learning rate η=0.0001. We repeat the above process until all the packages have completed. We refer to this process as an epoch. Then, we repeat the above process until the loss function stops converging or reaches the maximum number of epochs.

### 3.3. Performance Evaluation

Four metrics were used to evaluate the performance of IDPR prediction [42]. These were sensitivity (Sens), specificity (Spec), balanced accuracy (BACC), and Matthews correlation coefficient (MCC). The related formulas are as follows:

(29) $$\begin{array}{cc} Sens & =\frac{TP}{TP+FN} \end{array}$$

(30) $$\begin{array}{cc} Spec & =\frac{TN}{TN+FP} \end{array}$$

(31) $$\begin{array}{cc} BACC & =\frac{1}{2}\left(\frac{TP}{TP+FN}+\frac{TN}{TN+FP}\right) \end{array}$$

(32) $$\begin{array}{cc} MCC & =\frac{\left(TP\times TN)-(FP\times FN\right)}{\sqrt{\left(TP+FP)(TP+FN)(TN+FP)(TN+FN\right)}}. \end{array}$$

We use TP, FP, TN, and FN to represent the number of true positives, false positives, true negatives, and false negatives, respectively. The values of MCC can be any number between −1 and 1. The prediction accuracy for both ordered and disordered residue increases as the MCC value becomes closer and closer to 1.

## 4. Experimental Results

**Figure 3:**
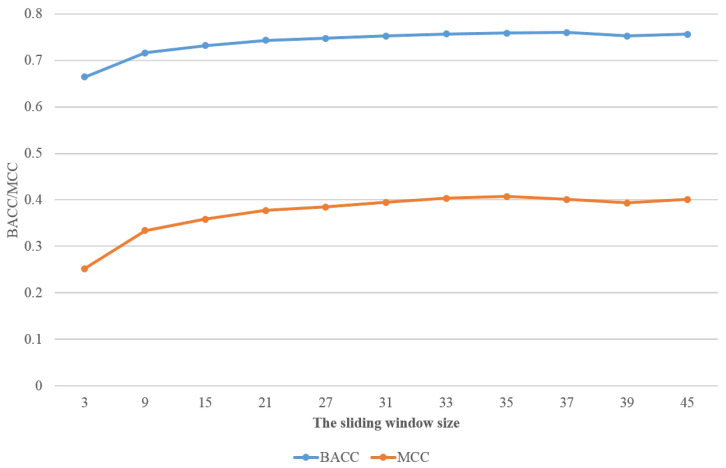
The performance with different sliding window sizes on BACC and MCC.

In this section, we will demonstrate the performance of our deep neural network on the different test sets: DIS166 [34], R80 [25], and MXD494 [33]. As a comparison, we also present the simulation results of the best known predictors for these datasets, such as RFPR-IDP (available at http://bliulab.net/RFPR-IDP/server (accessed on 26 March 2021)), SPOT-Disorder2 (available at https://sparks-lab.org/server/spot-disorder2/ (accessed on 26 March 2021)), DISvgg [16], and IDP-Seq2Seq [18]. For convenience, we refer to our method as MLP-VGG19-MLP. A ten-fold cross validation was performed on the training dataset DIS1450. The results of MLP-VGG19-MLP with different window sizes are shown in Table 1. In addition, the values achieved for BACC and MCC with different sliding window sizes are shown in Figure 3. When the sliding window size was larger than 33, the values tended to be smooth. Thus, we used the sliding window size of N=33 in subsequent simulations.

On the test sets DIS166, R80, and MXD494, the performance of MLP-VGG19-MLP was superior to that of RFPR-IDP, SPOT-Disorder2, and DISvgg. The MCC value of MLP-VGG19-MLP is 0.5674 on the test set DIS166, 0.5775 on the blind test set R80, and 0.4737 on the blind test set MXD494. The simulation results show that MLP-VGG19-MLP either considerably outperforms these methods or, when relying on a much smaller training dataset compared to the one used in [18], attains a performance similar to that of IDP-Seq2Seq [18]. Table 2, Table 3 and Table 4, respectively, present the performances of all these methods on test sets DIS166, R80, and MXD494.

## 5. Conclusions

In this study, a deep neural structure is developed for identifying IDPRs, where a variant VGG19 is situated between two MLP networks. Furthermore, for the first time, three novel sequence features—i.e., persistent entropy, PCAA2, and PCAA3—are introduced for identifying IDPRs. In comparison with our previous DISvgg algorithm, the prediction performance of MLP-VGG19-MLP exceeded it. Furthermore, only one VGG19 was used in this paper, while ten VGG16nets were employed in the previous paper. In comparison with RFPR-IDP, SPOT-Disorder2, and IDP-Seq2Seq, MLP-VGG19-MLP relies on a much smaller training set to achieve a performance that is better or similar to that achieved using other methods. The simulation results show that our neural structure either considerably outperforms other known methods or, when relying on a much smaller training set, attains a similar performance. Three novel sequence features could be used as valuable sequence features in the further development of identifying IDPRs.

## Figures and Tables

**Table 1:** Performance on dataset DIS1450 with different sliding window sizes.

Sliding Window Sizes	*Sens*	Spec	BACC	MCC
3	0.7471	0.5813	0.6642	0.2519
9	0.7972	0.6536	0.7164	0.3339
15	0.8192	0.6447	0.7319	0.3583
21	0.8183	0.6675	0.7492	0.3772
27	0.8233	0.6717	0.7475	0.3848
31	0.8183	0.6872	0.7527	0.3949
33	0.8125	0.7010	0.7568	0.4033
35	0.8100	0.7069	0.7585	0.4070
37	0.8679	0.6515	0.7597	0.4009
39	0.8266	0.6788	0.7527	0.3936
45	0.8214	0.6910	0.7562	0.4008

**Table 2:** Performance of various methods on dataset DIS166.

Methods	Sens	Spec	BACC	MCC
MLP-VGG19-MLP	0.8351	0.8338	0.8345	0.5674
DISvgg	0.6713	0.8828	0.7710	0.5132
RFPR-IDP	0.7557	0.7817	0.7687	0.4406
SPOT-Disorder2	0.7103	0.8084	0.7594	0.4952
IDP-Seq2Seq	0.7890	0.8212	0.8051	0.5475

**Table 3:** Performance of various methods on blind test dataset R80.

Methods	Sens	Spec	BACC	MCC
MLP-VGG19-MLP	0.7269	0.9261	0.8265	0.5775
DISvgg	0.5993	0.9429	0.7711	0.5270
RFPR-IDP	0.5464	0.9546	0.7505	0.5139
SPOT-Disorder2	0.4941	0.9439	0.7190	0.4486
IDP-Seq2Seq	0.7787	0.9124	0.8456	0.5884

**Table 4:** Performance of various methods on blind test dataset MXD494.

Methods	Sens	Spec	BACC	MCC
MLP-VGG19-MLP	0.7169	0.8081	0.7625	0.4737
DISvgg	0.7160	0.7956	0.7558	0.4577
RFPR-IDP	0.7490	0.7580	0.7540	0.4420
SPOT-Disorder2	0.6380	0.8200	0.7290	0.4482
IDP-Seq2Seq	0.7430	0.7910	0.7670	0.4750

## Data Availability

The datasets DIS1616, DIS1450, and DIS166; models; and code can be found at the website https://github.com/ZakeWang/MLP_VGG19_MLP.git accessed on 9 January 2022.
